# Phospholipid scramblase 1: a protein with multiple functions via multiple molecular interactors

**DOI:** 10.1186/s12964-022-00895-3

**Published:** 2022-06-01

**Authors:** Jessica Dal Col, Marìa Julia Lamberti, Annunziata Nigro, Vincenzo Casolaro, Elisabetta Fratta, Agostino Steffan, Barbara Montico

**Affiliations:** 1 Department of Medicine, Surgery and Dentistry “Scuola Medica Salernitana”, University of Salerno, Baronissi, Italy; 2 INBIAS, CONICET-UNRC, Río Cuarto, Córdoba, Argentina; 3 Division of Immunopathology and Cancer Biomarkers, Centro Di Riferimento Oncologico di Aviano (CRO), IRCCS, Aviano, Italy

**Keywords:** PLSCR1, Cell death, Protein–protein interaction, Inflammation, Virus

## Abstract

Phospholipid scramblase 1 (PLSCR1) is the most studied protein of the scramblase family. Originally, it was identified as a membrane protein involved in maintaining plasma membrane asymmetry. However, studies conducted over the past few years have shown the involvement of PLSCR1 in several other cellular pathways. Indeed, PLSCR1 is not only embedded in the plasma membrane but is also expressed in several intracellular compartments where it interacts with a diverse repertoire of effectors, mediators, and regulators contributing to distinct cellular processes. Although most PLSCR1 interactors are thought to be cell-type specific, PLSCR1 often exerts its regulatory functions through shared mechanisms, including the trafficking of different molecules within intracellular vesicles such as endosomes, liposomes, and phagosomes. Intriguingly, besides endogenous proteins, PLSCR1 was also reported to interact with exogenous viral proteins, thereby regulating viral uptake and spread. This review aims to summarize the current knowledge about the multiple roles of PLSCR1 in distinct cellular pathways.

**Video Abstract**

**Supplementary Information:**

The online version contains supplementary material available at 10.1186/s12964-022-00895-3.

## Background

Phospholipid scramblase 1 (PLSCR1) is to date the most extensively studied member of the scramblase family [1, 2].

The *MmTRA1b/*Plscr1 gene was originally cloned from a normal mouse kidney cDNA library and identified as the noncancerous counterpart of the leukemogenesis-associated gene *MmTRA1a, *which has been described for the first time by Kasukabe et al*.* in a mouse monocytic cell line [3–5]. Thereafter, the same group cloned the human homolog of mouse *MmTRA1b/Plscr1* [3]. PLSCR1 was characterized in 1996 in erythrocytes as a protein involved in the redistribution of phospholipids across the plasma membrane in response to an increase of intracellular calcium (Ca^2+^) [6]. In physiological conditions, the plasma membrane of erythrocytes, platelets and other cells is asymmetrical, whereby amino phospholipids, *i.e.*, phosphatidylserine (PS) and phosphatidylethanolamine (PE), are located within the inner membrane facing the cytoplasm and neutral polar phospholipids, *i.e.*, phosphatidylcholine (PC) and sphingomyelin (SM), reside in the outer leaflet at direct contact with the extracellular milieu. In that study Basse and colleagues purified and reconstituted the erythrocyte membrane elements and identified PLSCR1, a single polypeptide of approximately 37 kDa, as one of the main components involved in maintaining membrane asymmetry [6]. Conversely, in injured cells, scramblase activation promotes PS and PE redistribution to the outer leaflet of the plasma membrane, thus allowing cell recognition and elimination by phagocytes.

The PLSCR family consists of four homologous proteins, named PLSCR1-4. PLSCR1, PLSCR3, and PLSCR4 are ubiquitously expressed, whereas PLSCR2 expression is restricted to the testis. Human *PLSCR1* is clustered on the long arm of chromosome 3 (locus 3q24) together with *PLSCR2* and *PLSCR4*, whereas *PLSCR3* is located on chromosome 17. The *PLSCR1* gene consists of nine exons and the coding region spans from exon 2 through the end of exon 9 [7].

*PLSCR1* is a highly inducible gene. In particular, type I and II interferons (IFNs) are the most potent inducers of PLSCR1 expression [8–10]. Zhou et al*.* demonstrated that IFN-α promotes the transcription of this gene in a variety of human cell lines [10]. The analysis of *PLSCR1* sequence led to the identification of three putative IFN-regulated elements, of whom a single IFN-stimulated response element (ISRE), located in the untranslated exon 1, was found to be essential for IFN-dependent *PLSCR1* transcription [10]. IFN-induced *PLSCR1* expression is dependent on the activation of signal transducer and activator of transcription 1 (STAT1) [11] and also requires the sequential activation of protein kinase Cδ (PKCδ) and c-Jun N-terminus kinase (JNK) [11].

In silico studies of the *PLSCR1* promoter identified many additional putative sites for the transcription factors Snail, Sox-5, AML-1, HFH-3, Atbh1, Broad Complex, HNF-3b, Myf, Max, Spz-1, CREB, TBP, c-Fos, c-Rel, NF-kB, E2F, and c-Myc. In particular, Vinnakota et al*.* showed in mutagenesis experiments that two c-Myc binding sites are necessary for *PLSCR1* expression [12], whereas Francis et al*.* showed that Snail acts as both a transcriptional and translational repressor of *PLSCR1* expression in ovarian cancer cells [13].

PLSCR1 is a type II plasma membrane protein tail-anchored to the membrane through its C-terminus, while its long N-terminal domain is exposed to the cytoplasm. The proline-rich N-terminal region contains multiple PXXP and PPXY domains, a conserved cysteine rich domain, a Ca^2+^ binding EF-hand-like domain, a single transmembrane domain, a DNA binding domain [14, 15], and a nuclear localization signal [16]. The presence of these different domains and, particularly, of the DNA binding domain and of a nuclear localization motif, suggests that PLSCR1 is not only expressed within the plasma membrane and that it might exert different functions beyond the maintenance of phospholipid redistribution. Accordingly, molecular studies conducted in the last few years have characterized PLSCR1 as a key factor not only in cellular pathways at the plasma membrane level, but also within the nucleus, including the rRNA transcription control [17], the oxidative stress response [18], the regulation of cancer cell survival and proliferation [19–21], the autophagy process [22–24], and antiviral responses [25–32].

Based on these considerations, this review provides a comprehensive overview of the multiple functions of PLSCR1 in different cellular processes, with a particular focus on its molecular interactors. Nevertheless, the molecular mechanisms underlying PLSCR1 functions have yet to be fully characterized and more studies are awaited to better define its place in the mechanisms regulating cellular homeostasis and disease.

## PLSCR1 interactions with endogenous proteins

It is now well established that PLSCR1 is not only a membrane protein involved in phospholipid redistribution, but also contributes to different cellular processes by interacting with distinct molecules. Several studies have demonstrated the ability of PLSCR1 to directly bind to proteins located in both the cytoplasm (Table 1) and the nucleus (Table 2). Herein we describe PLSCR1 interactions with mediators participating in such cellular processes as cell death and autophagy, inflammation, molecular trafficking, and gene regulation.

**Table 1:** PLSCR1 interactions in the cytoplasm

Cellular processes affected	Protein	Disease	Study models	Methods	Effects of PLSCR1 interaction	References
Cell death	TRPC5	Cerebral ischemia–reperfusion injury	- HEK-293 - mouse Cortical neurons	- CO-IP - FRET ASSAY - PLA	Favors PS exposure and apoptosis induction	[35]
	PR3	Granulomatosis with polyangiitis	- Neutrophils - RBL-2H3	- CO-IP - IF	Induces PR3 externalization in plasma membrane and inhibition of apoptotic cell clearance by macrophages	[72]
	RELT	N.A	HEK-293	- Yeast two-hybrid screening -CO-IP	Favors RELT-induced cell death	[52]
Proliferation	ONZIN	N.A	- Myeloid cells - fibroblasts	- Yeast two-hybrid screening - CO-IP	Downregulates onzin effects on cell growth and proliferation	[21]
	SHC	N.A	- A431 - MEF - KEC	CO-IP	Promotes Src kinase activation through the EGF receptor	[56]
Autophagy	ATG12	MCL	- Mino - SP53 - Jeko-1	CO-IP	Prevents ATG16L1 recruitment thus inhibiting autophagy	[23]
	AIF	Leukemia	- NB4 - Jurkat	CO-IP	Interferes with ATG12/ATG5 complex inhibiting autophagy	[24]
Egf-induced cell responses	EGFR (LIPID RAFTS)	N.A	- Human oral epithelial - Carcinoma KB cells	CO-IP	Contributes to EGFR trafficking	[55]
Mast cell degranulation	LYN SYK (LIPID RAFTS)	N.A	- HEK-293 - RBL-2H3	CO-IP	Modulates the LAT/PLCγ1/Ca^2+^ axis, thus resulting in reduced degranulation and VEGF production	[69]
β-Peptide formation	BACE	Alzheimer's disease	- HeLa - HEK-293 - SH-SY5Y	- Yeast two-hybrid screening - CO-IP	Regulates intracellular trafficking of BACE and regulation of amyloid β-peptide formation	-[63]
TLR9-mediated DNA sensing	TLR9	N.A	- Human pDC - HEK-293 T	Yeast two-hybrid screening	Necessary for the nuclear translocation of IRF7 and IFN-α production following CpG-A stimulation	[57]

*N.A.* Not applicable, *TRPC*5 Transient receptor potential canonical 5, *CO-IP* Co-immunoprecipitation, *FRET* Förster Resonance Energy Transfer, *PLA* proximity ligation assay, *PLSCR*1 phospholipid scramblase 1, *PS* phosphatidil-serin, *RELT* Receptor expressed in lymphoid tissues, *PR*3 Proteinase 3, *ATG*12 autophagy-related protein 12, *MCL* Mantle cell lymphoma, *AIF* apoptosis-inducing factor, *EGFR* epidermal growth factor receptor, *LAT* Linker For Activation Of T Cells, *PLC*γ1 phospholipase Cγ1, *BACE* β-Site amyloid precursor protein (APP)-cleaving enzyme, *TLR*9 Toll-like receptor 9, *IF* Immune fluorescence, *IFN*-α interferon-alpha, *IRF*7 IFN regulatory factor 7, *CpG-A* A-type CpG DNA

**Table 2:** PLSCR1 interactions in the nucleus

Cellular processes affected	Protein	Disease	Study models	Methods	Effects of PLSCR1 interaction	References
Nuclear import	Importin-α	N.A	Murine SVT2 fibroblasts	- Fluorescence Anisotropy Assay - Crystallographic Analysis	Favors PLSCR1 entrance into the nucleus	[16]
- Cell migration - Invasion - Stemness	STAT3	BLBC	- MDA -MB231 - SUM159 - MCF7 - HCC1937 - T47D - MDA -MB468	- MS - CO-IP	Activates STAT1 transcription thus inducing cancer stem cell properties	[81]
Cell differentiation	IP3R1 promoter	AML	Primary leukemia cells	EMSA	Induces cell cycle arrest and cell differentiation promoted by Wogonoside	[43, 77]
Cell signaling	TOPO IIα TOPO IIβ	N.A	HeLa	- Yeast two hybrid screening - GST-pull-down assay - CO-IP	Increases DNA decatenating ability of Topo IIα	[15]
Cell proliferation	ANG MK	N.A Hepatic carcinoma	HeLa HepG2	- Yeast two hybrid screening - GST-pull-down assay - CO-IP - FRET	Positively regulates rRNA transcription	[83]
				- GST-pull-down assay - CO-IP	Promotes cell proliferation and migration	[80]

*N.A.* Not applicable, *PLSCR*1 Phospholipid scramblase 1, *BLBC* Basal-like breast cancer, *STAT*3 Signal Transducer And Activator Of Transcription 3, *STAT*1 Signal Transducer And Activator Of Transcription 1, *MS* Mass spectrometry, *CO-IP* co-immunoprecipitation, *IP3R*1 1,4,5-trisphosphate receptor 1, *AML* acute myeloid leukemia, *EMSA* Electrophoretic mobility shift assay, *TOPO* II DNA topoisomerase II, *ANG* Angiogenin, *FRET* fluorescent resonance energy transfer, *MK* Midkine

### PLSCR1 interactions with cell death-related proteins

**Fig. 1:**
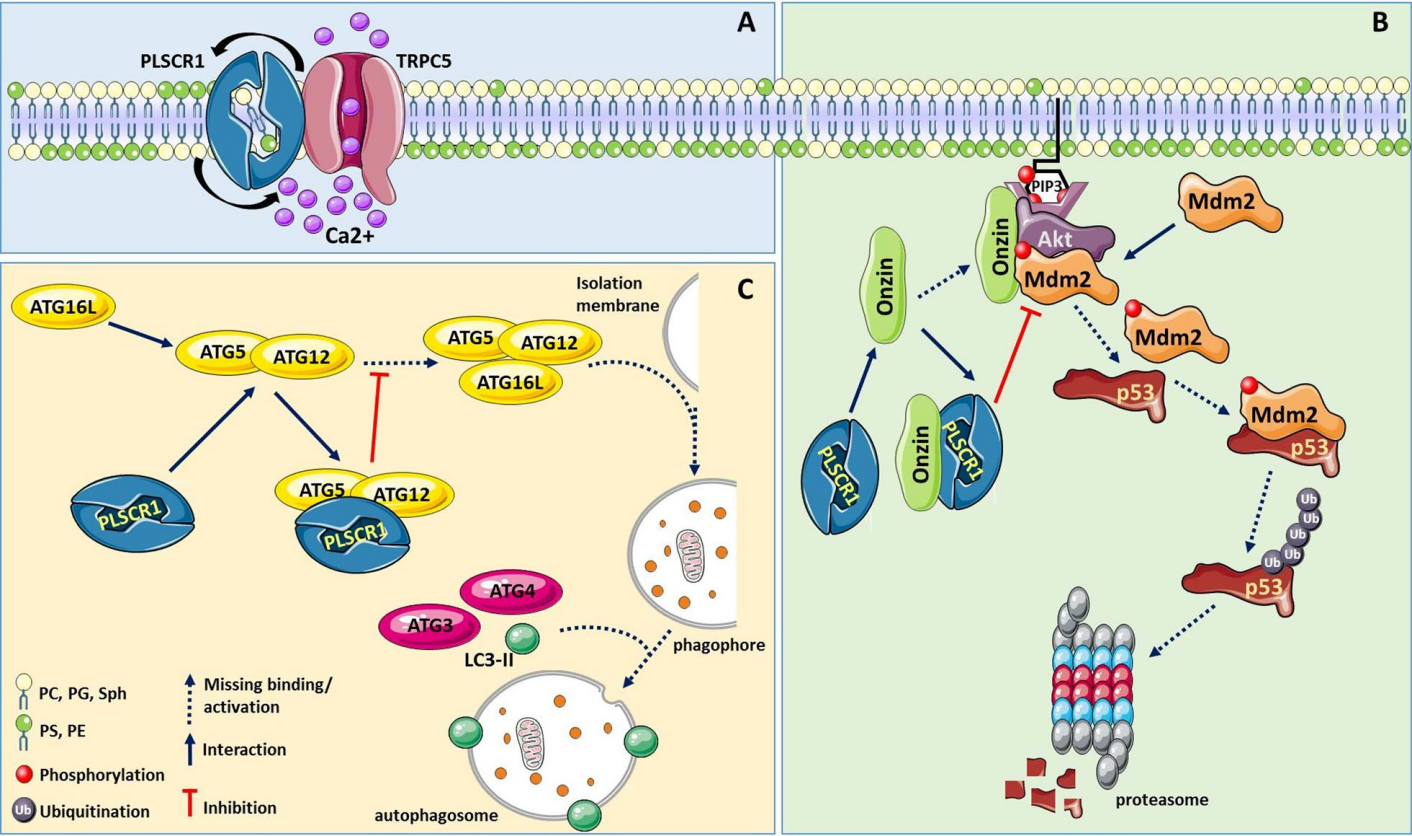
Main molecular mechanisms linking PLSCR1 protein to apoptotic and autophagic processes. **A** Transient receptor potential canonical 5 (TRPC5) Ca^2+^ channel directly interacts with PLSCR1 and through Ca^2+^ influx activates it, promoting phosphatidylserine (PS) exposure on the outer leaflet of the cell membrane. **B** PLSCR1 favours p53-dependent apoptosis by association with the protein onzin, the resulting inhibition of Mdm2 phosphorylation/activation by Akt, and thus preserving p53 from proteasome degradation. **C** The ability of PLSCR1 to bind the complex ATG5/ATG12 complex, replacing the third member ATG16L, inhibits the elongation of the phagophore and autophagosome generation blocking the autophagic process. When autophagy plays an anti-apoptotic role through this mechanism, PLSCR1 promotes programmed cell death

PS exposure during apoptosis acts as an “eat me” signal, which leads to PS-dependent engulfment of dead cells by phagocytes [33]*.* While earlier studies reported the involvement of PLSCR1 in apoptosis-associated PS externalization [34, 35], only the recent work by Guo et al*.* detailed the mechanisms underlying PLSCR1-promoted PS exposure [35]. Specifically, they demonstrated a direct interaction between the C-terminal domains of PLSCR1 and of the transient receptor potential canonical 5 (TRPC5). TRPC5 is a Ca^2+^-permeable channel and its involvement in apoptosis induction was recently demonstrated in neuronal cells [36]*.* Its interaction with PLSCR1 was found to be required for PSLCR1 to induce PS exposure on the outer leaflet of the plasma membrane. In these studies, TRPC5 activated PLSCR1 scrambling activity via the influx of Ca^2+^ from the extracellular milieu [35], and TRPC5 knockout resulted in reduced PS exposure in neurons (Fig. 1A) [35].

Besides its role in apoptosis, PS exposure is a key regulatory event of the coagulation cascade since it serves as a catalytic surface for the assembly of coagulation factors. PLSCR1 expression was significantly upregulated in whole blood samples and peripheral blood mononuclear cells from patients with systemic lupus erythematosus (SLE), a chronic autoimmune disease with multiple organ involvement, in which autoantibodies and immune complexes induce tissue damage [37]. As expected, heightened expression of PLSCR1 and increased PS exposure were associated with increased fibrin turnover, which may explain, at least in part, the thrombophilia commonly reported in SLE patients [38]. An increase in PS scrambling to the outer membrane in erythrocytes has also been associated to other pathologies, such as diabetes and hypertension, which appears to be regulated by tubulin content and distribution and flippase activity [39]. Further studies should be conducted to elucidate the specific impact of PLSCR1 on this phenomenon.

Besides promoting PS exposure, PLSCR1 may affect apoptosis through other mechanisms involved in cancer progression. For instance, in myeloid cells, PLSCR1 interacts with the c-Myc target onzin [21], a protein that has been reported to promote cell survival and proliferation in specific cancer lineages [40]. Onzin has been involved in adipocyte growth [9, 41] and in the progression of leukemia [24, 42–44], hepatocellular carcinoma [45], pancreatic cancer [46, 47], and colon cancer [19, 20, 48]. As described by Li et al*.*, onzin may interact with and activate Akt1 and Mdm2, thus resulting in loss of p53-dependent cell cycle arrest and apoptotic response in myeloid cells. In the same study, it was demonstrated that PLSCR1 acts as a negative regulator of onzin [21] by competing with Akt1 and Mdm2 (Fig. 1B). Hence, PLSCR1 depletion in myeloid cells, normally expressing onzin, resulted in a phenotype that mimics onzin over-expression, whereas PLSCR1 expression could restore p53 induction and cell cycle arrest in response to apoptotic stimuli [21].

The involvement of PLSCR1 in apoptosis control occurs also through the interaction with the receptor expressed in lymphoid tissues (RELT). RELT is a tumor necrosis factor (TNF) receptor that is particularly abundant in hematologic tissues and peripheral blood leukocytes as well as leukemias and lymphomas [49]. This protein can activate NF-κB, p38 and JNK via binding to the SPS1-related proline/alanine-rich kinase (SPAK), a close homolog of oxidative-stress responsive 1 (OSR1) [50]. Intriguingly, Cusick et al*.* showed that PLSCR1 could interact with all three members of the RELT family and showed that RELT could simultaneously bind to OSR1 and PLSCR1; in fact the formation of this complex allowed PLSCR1 phosphorylation by OSR1. Moreover, overexpression of RELT induced PLSCR1 subcellular redistribution from the plasma membrane to the perinuclear region where these two proteins co-localized [18]. In HEK-293 cells the overexpression of RELT molecular species or PLSCR1 resulted in increased cell death rate subsequent to activation of the apoptotic pathway [51]. Notably, concomitant overexpression of RELT and PLSCR1 did not have any additional or synergic effect on the promotion of cell death, indicating that the two proteins are likely involved in the same pro-apoptotic pathway [52]. However, the precise molecular mechanism underlying RELT/PLSCR1 interaction has yet to be elucidated.

When tumor cells are exposed to pro-apoptotic treatments, dying cells often activate autophagy in an attempt to survive. Autophagy is a self-degradative process aimed at preventing the initiation of the cell death program. By exploiting a novel hybrid yeast-human network analysis, Huett et al*.* identified PLSCR1 as one of the binding partners of autophagy-related protein (ATG) 12 [53]. ATGs include two ubiquitin-like proteins, ATG12 and ATG8, both involved in the formation of autophagosomes, transient organelles specialized in the sequestration and the lysosomal or vacuolar transport of cellular constituents. During autophagy, ATG12 undergoes conjugation to ATG5; the ATG12/ATG5 complex is essential for membrane structure expansion during phagophore formation [22, 54]. Subsequently, the ATG12/ATG5 complex further interacts with ATG16L1. Phagophore expansion and cargo recruitment are carried out by ATG4 and ATG3 that promote the conjugation of PE to microtubule-associated protein light chain 3 (LC3), forming lipidated LC3-II, in order to permit proper autophagosome formation and its fusion with lysosome. In the resulting autolysosome the cargo is finally degraded. PLSCR1 was found to interact with the ATG12/ATG5 complex, thus preventing ATG16L1 recruitment and its full activation (Fig. 1C). Moreover, upon autophagy stimulation, PLSCR1 can be transferred into the autophagosome/autolysosome where the protein co-localizes with LC3-II puncta. PLSCR1 overexpression inhibited the autophagic process possibly by interfering with phagophore elongation, thus preventing the correct completion of autophagy [22, 23]. Notably, Shi et al*.* demonstrated that p53 status might affect autophagy and apoptosis through the modulation of PLSCR1 expression and its association with different interactors. In this study, wild-type p53 induced PLSCR1 transcription in leukemia cells treated with sodium selenite, concomitant with apoptosis induction and autophagy inhibition. In contrast, in the presence of mutated p53, sodium selenite treatment failed to up-regulate PLSCR1. In addition, PLSCR1 inhibitory effect on autophagy was prevented through its sequestration by apoptosis-inducing factor (AIF), which interfered with PLSCR1 binding to the ATG12/ATG5 complex [24].

Altogether, these data clearly indicate that PLSCR1 contributes to the induction of programmed cell death either directly, by promoting PS exposure, or indirectly by interacting with both positive and negative modulators of this process, from case to case enhancing or inhibiting their functions, respectively. Therefore, these findings indicate that PLSCR1 may exhibit an anti-tumor function by promoting apoptosis. However, as discussed below, controversial evidence suggests that PLSCR1 may have both anti- and pro-survival roles in neoplastic malignancies.

### PLSCR1 interactions with membrane receptors

As a transmembrane protein, PLSCR1 was also described as a component of membrane lipid rafts. Sun et al*.* demonstrated that PLSCR1 could physically and functionally interact with epidermal growth factor receptor (EGFR) and the adapter molecule Shc in human oral epithelial carcinoma cells [55]. Stimulation with EGF increased the expression of PLSCR1 and induced the internalization of both EGFR and PLSCR1 and their trafficking into endosomal pools. Whereas EGFR was ultimately degraded, most of the endocytosed PLSCR1 was recycled to the cell surface. Detailed mechanistic studies revealed that, after EGF stimulation, PLSCR1 required phosphorylation at both Tyr69 and Tyr74 by Src kinase in order to bind Shc [56]. Of interest, PLSCR1 depletion resulted in decreased activation of Src following exposure to growth factors, suggesting that PLSCR1 might favor receptor-dependent activation of Src. Therefore, PLSCR1 may regulate EGFR signaling by contributing to receptor trafficking in the intracellular membranes and/or by controlling the posttranscriptional effector pathways mediating the cellular response to EGF [55, 56].

In a study by Talukder et al*.*, PLSCR1 was also implicated in Toll-like receptor 9 (TLR9) trafficking to the early endosome in plasmacytoid dendritic cells (pDCs) [57], a minor population of circulating dendritic cells (DCs) that produces large amounts of type I IFNs. TLR9 is an endosomal receptor specialized in sensing viral DNA through the detection of CpG motifs (unmethylated CG dinucleotides in certain base contexts) and is selectively expressed by pDCs and B lymphocytes. When cells perceive nucleic acids, TLR9 moves from the endoplasmic reticulum to endosomes where it interacts with foreign DNA [58]. Interestingly, Talukder et al*.* reported that PLSCR1 could bind to TLR9 through the proline-rich G-box-binding domain before TLR9 entry into the endosome and, although TLR9 is generally cleaved to generate the functional C-terminal receptor, PLSCR1-TLR9 association did not require the activating cleavage of the receptor [57]. Current DC-based immunotherapeutic approaches exploit synthetic ligands of TLR9, that is, CpG motif-containing oligodeoxynucleotides (CpG ODN), to help differentiate/activate DCs. In a pilot study Krieg et al*.* showed that TLR9 stimulation with one class of CpG ODN, named CpG-A, induced high levels of IFN-α secretion [59]. Interestingly, Talukder et al*.* demonstrated that the interaction between PLSCR1 and TLR9 was selectively required for IFN-α production upon CpG-A stimulation, whereas it was dispensable for secretion of such other cytokines as IL-6 and TNF-α [57]. Moreover, the presence of PLSCR1 influenced IFN regulatory factor (IRF) 7 nuclear translocation following TLR9 pathway activation. PSLCR1-effected control of TLR9 signaling through the regulation of its trafficking suggests the potential involvement of this protein in antiviral innate immune responses and in self-nucleic acid-associated autoimmune diseases. In line with this consideration, and with findings reported in the above section, different studies reported the possible involvement of PLSCR1 in SLE pathogenesis [37, 38, 60–62]. Consistently, multi-dataset analyses identified PLSCR1, along with nine other genes, as a putative common biomarker for SLE and the biological processes most significantly enriched in association with these genes were “response to virus” and “immune response” [37]. Moreover, hypomethylation of four IFN-responsive genes, including PLSCR1, was found by Yeung et al*.* in patients with SLE using genome-wide DNA methylation microarray and bisulfite pyrosequencing [62]. However, more studies are needed to fully understand the mechanisms of PLSCR1 involvement in SLE pathogenesis besides the above-mentioned function in PS scrambling.

As reported by Kametaka et al., PLSCR1 can also control intracellular trafficking of membrane proteins in neuronal cells [63]. In fact, PLSCR1 has been described as a binding partner of the β-secretase β-site amyloid precursor protein (APP)-cleaving enzyme (ΒACE), a membrane proteinase that processes the APP to generate the neurotoxic amyloid β-peptide. Mutations of the APP gene are associated with Alzheimer’s disease (AD), in which the amyloid β-peptide is the principal constituent of the senile plaques, the major AD hallmark [64, 65]. In neuronal cells, BACE β-secretase activities occur at least in three sites including the endoplasmic reticulum, the Golgi/trans Golgi network and the endosomal compartments. Recently, Ito K et al*.* demonstrated that a glutamatergic receptor antagonist, widely used as medication for the treatment of AD, leads to reduced amyloid β-peptide production and plaque deposition by impairing the intracellular trafficking of the BACE substrate APP [66]. On these grounds, how the interaction between PLSCR1 and BACE may affect BACE intracellular trafficking acquires particular relevance. The interaction between the two proteins requires a di-leucine repeat in BACE that is necessary for its endocytic transport [63]. This suggests that PLSCR1, by controlling the intracellular trafficking of BACE, might interfere with amyloid β-peptide formation. However, no data have yet been published in support of this notion and further studies are needed to more thoroughly investigate the role of PLSCR1 in AD.

Taken together, these findings clearly indicate that PLSCR1 plays a key role in modulating intracellular trafficking of receptors and membrane proteins by interacting with different molecules in a cell- and/or disease-specific context.

### PLSCR1 interactions with inflammatory factors

**Fig. 2:**
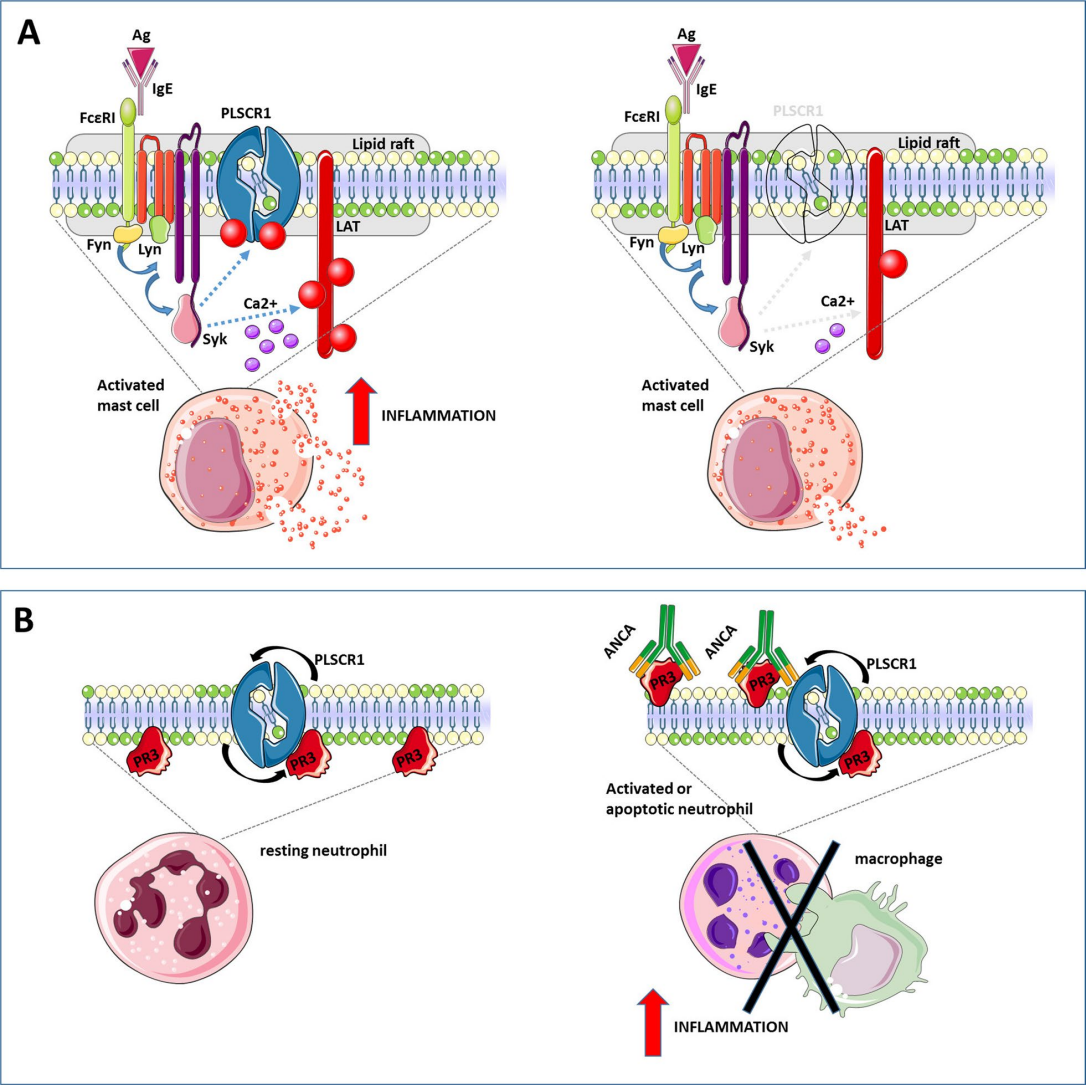
PLSCR1 role in inflammation. **A** In the activated mast cells, following FcεRI aggregation after antigen capture by IgE bound to the receptor, different tyrosine kinases are activated, which can in turn phosphorylate PLSCR1 and several signaling intermediates ending into the mobilization of Ca^2+^. Finally, FcεRI activation and PLSCR1 phosphorylation result in increased degranulation and inflammation. **B** PLSCR1 also plays a pro-inflammatory role in neutrophils. PLSCR1 contributes to proteinase 3 (PR3) externalization on the plasma membrane and neutrophil activation. PR3 is the preferred target of anti-neutrophil cytoplasm autoantibodies (c-ANCA), whose binding to PR3 interferes with apoptotic neutrophil clearance by macrophages promoting inflammation

PLSCR1 is recruited to lipid rafts in activated mast cells where it was found to co-localize with the kinases Lyn and Syk, leading to tyrosine phosphorylation of PLSCR1 [67]. Lyn and Syk are implicated in signaling from the high-affinity receptor for immunoglobulin E (FcεRI), which is constitutively expressed on mast cells and is involved in the immune responses to parasites and allergens. Aggregation of FcεRI results in the activation of such tyrosine kinases as Lyn, Fyn and Syk and sequential phosphorylation of several intermediate molecules, among which the linker for activation of T-cells (LAT), thus resulting in the mobilization of Ca^2+^. FcεRI-dependent activation of mast cells finally results in the release of inflammatory mediators stored in cytoplasmic granules through a degranulation process [68]. Interestingly, PLSCR1 knockdown in mast cells decreased the degranulation response to FcεRI aggregation and the release of vascular endothelial growth factor (Fig. 2A). This was associated with reduced phosphorylation of LAT and inhibition of Ca^2+^ mobilization [69, 70]. Accordingly, Kassas-Guediri et al*.* demonstrated that PLSCR1 can amplify the anaphylactic response in vivo by augmenting IgE/antigen-induced mast cell degranulation [71]. Therefore, PLSCR1 may critically contribute to FcεRI-mediated immune responses by promoting the release of pro-inflammatory mediators and cytokines.

PLSCR1 pro-inflammatory properties have also been involved in granulomatosis with polyangiitis (GPA; formerly known as Wegener’s granulomatosis), a rare chronic, potentially fatal, autoimmune disease characterized by blood vessel inflammation and the formation of necrotizing granulomas [72]. A study by Kantari et al*.* showed that the externalization of proteinase 3 (PR3) on the plasma membrane of apoptotic neutrophils in this condition was dependent on interaction with PLSCR1 and that ecto-PR3 inhibited the clearance of these cells by macrophages [72]. PR3 is the preferential target of anti-neutrophil cytoplasm autoantibodies (c-ANCA) in GPA and belongs to the family of neutrophil microbicidal serine proteinases that are stored in azurophilic granules. PR3 is expressed at the inner plasma membrane of resting neutrophils and its exposure on the outer leaflet increases upon cell activation and during apoptosis. The binding of c-ANCA to neutrophil expressing surface PR3 results in their activation. PR3 also interacts with PS, a major component of neutrophil-derived microvesicles (MVs) [73]. These MVs are thought to contribute to the oxidative damage of endothelial cells and the systemic inflammatory response seen in patients with GPA. While PR3-expressing neutrophils produce fewer MVs following activation and apoptosis, PLSCR1 may contribute to the pro-inflammatory effects of PR3 externalization by favoring its binding by c-ANCA and interfering with apoptotic neutrophil clearance by macrophages (Fig. 2B).

Due to its roles in promoting inflammatory events, PLSCR1 expression might have major implications in immune regulation. Consistently, Herate et al*.* showed that PLSCR1 depletion stimulated FcR-mediated phagocytosis during monocyte-to-macrophage differentiation, whereas its overexpression inhibited phagocytosis in the monocytic cell line THP-1. In differentiated macrophages, PLSCR1 was recruited to phagocytic cups and remained associated with the phagosomes [74]. However, the precise molecular mechanisms and the PLSCR1 interactors involved in these processes remain unclear; likewise, the potential implications of PLSCR1-mediated negative regulation of phagocyte functions need to be addressed in more detail.

### PLSCR1 interactions with nuclear proteins

Although PLSCR1 is mainly located in the plasma membrane, it also contains a non-classical nuclear localization signal (NLS). The interaction between the NLS and the nuclear import carrier proteins importin-α/β [14, 16] allows PLSCR1 transfer into the nucleus [14, 75], where it directly binds genomic DNA. Nuclear localization of PLSCR1 is controlled by palmitoylation at a specific cysteine-rich sequence ^184^CCCPCC^189^. In addition, EGF signaling could induce PLSCR1 nuclear translocation through its phosphorylation at Tyr69 and Tyr74 [56].

Only sparse information is available about PLSCR1 nuclear interactome and the functional significance of these interactions is not completely clear. Within the nucleus, PLSCR1 was found to interact with topoisomerases (TOPO) II α and β and increase the DNA decatenating ability of TOPO II α [15], but to date no connection with specific cell functions has been documented. Nuclear PLSCR1 was also shown to enhance the expression of inositol 1,4,5-triphosphate receptor type 1 (IP3R1), a protein involved in regulating Ca^2+^ release from the endoplasmic reticulum [76]. A binding site for PLSCR1 was identified within the *IP3R1* promoter region (^−101^GTAACCATGTGGA^−89^) next to a binding site for the transcription factor AP-2, which regulates *IP3R1* transcription. However, it is not clear whether PLSCR1 activates directly *IP3R1* expression by acting as a transcription factor, or as a co-factor enhancing AP-2 transcriptional activity. Besides regulating its expression, PLSCR1 can directly interact with IP3R1. In particular, in acute myeloid cells PLSCR1/IP3R1 interaction has been involved in cell cycle arrest and cell differentiation promoted by the plant-derived flavonoid wogonoside [43, 77].

Interestingly, in basal-like breast cancer (BLBC) nuclear PLSCR1 was found to interact with STAT3 and bind to contiguous sites at the *STAT1* promoter region, resulting in *STAT1* transcriptional activation [78]. Notably, PLSCR1 overexpression has been specifically detected in BLBC relative to the luminal subtype. The BLBC subtype is usually associated with large tumor size, high grade, metastasis, early recurrence, and poor survival, and high-level expression of PLSCR1, contributing to enhanced *STAT1* activation, was found to promote cancer stem cell properties [78]. Furthermore, PLSCR1 knockdown resulted in reduced proliferation, invasion and migration in BLBC in vitro cultures, and reduced BLBC growth in vivo [78]. This PLSCR1-STAT3 axis was confirmed by Liao et al*.*, who demonstrated that karyopherin-α2 promoted radioresistance in lung adenocarcinoma by boosting PLSCR1-STAT3-mediated induction of STAT1-dependent signaling [79]. In a pilot study by Huang et al*.* PLSCR1 induced proliferation and migration of hepatic carcinoma cells through the interaction with the multifunctional growth factor midkine (MK) within the nucleus [80]. In line with these data, Gui et al*.* recently demonstrated that PLSCR1 expression is up-regulated in primary liver cancer and is associated with the clinical stage and hepatitis B virus (HBV) infection [81]. PLSCR1 knockdown significantly inhibited the proliferation, adhesion, migration and invasion of cancer cells, suggesting that PLSCR1 might be a suitable therapeutic target in regimens aimed at preventing the progression of primary liver cancers [81].

Nuclear PLSCR1 has also been shown to bind to angiogenin (ANG). This interaction was first identified through yeast two-hybrid screening, and thereafter confirmed by in vitro and in vivo studies [17]. ANG is a key protein implicated in angiogenesis in normal and tumor growth and is associated with cancer and neurological diseases. ANG accumulates in the nucleolus to enhance rRNA transcription, thus promoting cell proliferation [17, 82, 83]. In line with this, Zhu et al*.* demonstrated that ectopic expression of PLSCR1 enhanced rRNA transcription, but this effect was abolished after ANG depletion, thus indicating that PLSCR1 could positively regulate rRNA transcription by interacting with ANG [17]. These results might support a new function of PLSCR1 in angiogenesis, which should be further validated in suitable models.

Collectively, these findings suggest that the pro- or anti-tumor properties of PLSCR1 may depend on the intracellular locations of PLSCR1 and its interactors. In fact, whereas PLSCR1 interactions with cytoplasmic proteins, such as onzin, ATG12/ATG5 or AIF, may promote neoplastic cell death [21, 24], its nuclear association with STAT3 or MK would rather enhance cancer cell proliferation and progression [78, 80].

## PLSCR1 in viral infections

As discussed above, PLSCR1 expression is potently induced by IFNs, whose central roles in initiating immune responses, especially during viral infections, are well established. PLSCR1 involvement in IFN-initiated antiviral responses has been previously described [25]. While the previous paragraphs have outlined PLSCR1 interactions with endogenous proteins, here we aim to discuss the documented ability of PLSCR1 to bind exogenous proteins associated with viral species, from case to case resulting in antiviral or, less frequently, pro-viral functions (Table 3).

**Table 3:** PLSCR1 interactions with viral proteins

Virus	Protein	Study model	methods	Effects of PLSCR1 interaction	Pro/anti	References
HBV	HBx	- HEK-293 - HepG2 - Huh7	- Yeast two-hybrid screening - GST pull-down assay - CO-IP	Induces HBx ubiquitination and proteasome degradation	Anti virus	[32]
HIV	TAT	- COS-1 - MOLT/HIV	- Pull-down assay - CO-IP	Inhibits Tat functions by reducing its nuclear localization	Anti virus	[28]
	SLPI	- CD4^+^ HPB-ALL - Jurkat	- Yeast two-hybrid screening - GST pull-down assay - CO-IP - ELISA interaction test	Perturbs the virus entry process by modulating CD4-SLPI interaction	Anti virus	[85]
HTLV1	TAX	COS-1	- Pull-down assay - CO-IP	Reduces the cytoplasmic distribution of TAX and its homodimerization	Anti virus	[27]
IAV	NP	- A549 - HEK-293 T - THP-1 - U251	- Yeast two-hybrid screening - CO-IP	Inhibits the nuclear import of NP/vRNP thus limiting viral replication	Anti virus	[29]
EBV	BZLF1	- HEK-293 - COS-1 - HeLa - A431 - MCF-7 - SW480 - C666-1 - BJAB - B958 - Namalwa - P3HR1 - Daudi	- Pulldown assay - CO-IP	Represses BZLF1-dependent lytic gene expression	Anti virus	[87]
HCMV	CREB CBP IE2	- HEL - OUMS-36 T-3 - HEK-293	- Pull-down assay - CO-IP	Inhibits CREB functions and CREB-IE2, CBP-IE2 complexes, resulting in the repression of HCMV replication	Anti virus	[89]
HSV1 HSV2	Glycoprotein L	- CaSki - K2/E6E7 - Vero - HaCAT	- PLA - CO-IP	Favors AKT translocation to the outer leaflet of plasma membrane followed by glycoprotein B binding and viral entry	Pro virus	[90]
HCV	E1 E2 OCLN	- HEK-293 T - Huh7.5.1	- Yeast two-hybrid screening - Pull-down assay - CO-IP	Interacts with HCV envelope proteins and favors viral entry	Pro virus	[26]

*HBV* Hepatitis B virus, *HBx* HBV encoded X protein, *HCC* hepatocellular carcinoma, *CO-IP* Co-immunoprecipitation, *HIV-*1 human immunodeficiency virus type-1, *SLPI* Secretory leukocyte protease inhibitor, *HTLV*-1 Human T-cell leukemia virus type-1, *IAV* influenza A virus, *NP* nucleoprotein, *vRNP* viral ribonucleoprotein, *EBV* Epstein-Barr virus, *HSV* Herpes simplex virus, *PLA* Proximity ligation assay, *HCV* Hepatitis C virus, *OCLN* occluding, *HCMV* Human cytomegalovirus, *CREB* cAMP-responsive element-binding protein, *IE*2 HCMV immediate early protein 2, *CBP* CREB-binding protein

### Antiviral activity of PLSCR1

To dissect the specific signaling program activated by IFNs following viral infection, Mets et al*.* developed a RNA interference-based “gain of function” screen through which they identified many IFN-stimulated genes involved in the inhibition of hepatitis C virus (HCV) replication, including PLSCR1 [30]. Therefore, in HCV infection, PLSCR1 is thought to contribute to IFN antiviral function. However, PLSCR1 was also reported to promote HCV viral entry by interacting with the E1 and E2 HCV envelope proteins as well as with the entry factor occludin [26]. On the other hand, Yang et al*.* provided the first evidence that PLSCR1 inhibited HBV replication both in vitro and in vivo by reducing the expression of viral proteins, replicative intermediates, and total viral RNA. More specifically, ectopic expression of PLSCR1 in HBV-infected cells was sufficient to induce antiviral IFN signaling through the up-regulation of STAT1 and STAT2 [31]. Subsequently, Yuan et al*.* confirmed the important role of PLSCR1 in the host defense against HBV infection. In particular, they demonstrated that PLSCR1 promoted the ubiquitination and proteasomal degradation of the HBV X protein (HBx), resulting in reduced HBx levels [32]. More importantly, the same study documented that patients with low plasma levels of PLSCR1 were at higher risk to develop hepatocellular carcinoma [32].

PLSCR1 can also interfere with other viruses able to promote cancer development, including the human T-lymphotropic virus type 1 (HTLV-1), a virus known to cause adult T-cell leukemia/lymphoma and neurodegenerative disorders. Along this line, Kusano and Eizuru documented that PLSCR1 interacts with HTLV-1 transactivator (Tax), a protein necessary for provirus transcription. Tax localizes in both the nucleus and the cytoplasm and its subcellular distribution pattern is important for the transforming capabilities of the virus [84]. Notably, PLSCR1-Tax interaction reduced the cytoplasmic redistribution of Tax and its homodimerization, thus inhibiting Tax-mediated transactivation of HTLV-1 long terminal repeat [27].

PLSCR1 was also shown to physically interact with human immunodeficiency virus 1 (HIV-1) Tat protein that is required for efficient provirus transcription and HIV replication [28]. Kusano and collaborators demonstrated a direct interaction between PLSCR1 and Tat, which resulted in reduced Tat nuclear localization and functions [28]. On the other hand, Py et al*.* reported that PLSCR1 and its homolog PLSCR4 may interact with the cytoplasmic domain of CD4, the main T-lymphocyte receptor involved in HIV-1 entry. Moreover, PLSCR1 may act as a cytoplasmic receptor for secretory leucocyte protease inhibitor (SLPI), a polypeptide secreted by epithelial cells in mucosal fluids and exhibiting HIV-specific antiviral activity associated with perturbation of the virus entry process [85]. Of note, SLPI competed with CD4 for the same binding region on PLSCR1 [85], which suggests that PLSCR1 may interfere with the molecular mechanisms underlying the antiviral activity of SLPI. Nevertheless, how the PLSCR1/CD4/SLPI axis regulates HIV entry remains to be fully elucidated.

Distinct mechanisms are at play in the reported ability of PLSCR1 to inhibit replication of influenza A virus (IAV), the causative agent of influenza in many species. In this model, PLSCR1 was found to directly bind the viral nucleoprotein (NP) and interfere with its nuclear import [29]. NP is a component of the virus ribonuclear protein complex, which is responsible for the transcription and replication of the viral genome that takes place in the nucleus of infected cells [86]. Specifically, NP is responsible for the active nuclear import of the virus by forming a complex with the host importin-α. This interaction, and the ensuing nuclear translocation, are prevented contingent upon PLSCR1 binding, resulting in virus life cycle arrest [29].

More recently, Kusano and Ikeda discovered that PLSCR1 specifically interacts with the Epstein-Barr virus (EBV) immediate-early transactivator BZLF1 [87]. EBV is the etiologic agent of nasopharyngeal carcinoma (NPC) and is known to directly promote the neoplastic transformation of lymphoid cells, resulting in the development of a variety of lymphoproliferative disorders [88]. Consistently, the basal expression of PLSCR1 was significantly higher in EBV-infected NPC cells. Evidence from both in vitro and in vivo studies indicated that PLSCR1 and BZLF1 co-localize to the nucleus where PLSCR1 prevents the interaction between BZLF1 and the cAMP response element-binding protein (CREB) binding protein (CBP), which is required for efficient transactivation of EBV early promoters by BZLF1. Therefore, a high-level PLSCR1 expression might promote the switch from lytic to latent EBV infection that usually occurs in EBV-associated epithelial malignancies, including NPC [87].

Sadanari et al*.* have recently demonstrated that PLSCR1 is also implicated in the control of human cytomegalovirus (HCMV) infection. In this study, PLSCR1 could interact with CREB, HCMV immediate early protein 2 (IE2) and CBP, all of which are required for HCMV replication. In particular, the interaction between PLSCR1 and CREB was found to be critical for blocking HCMV infection, given the central role of this nuclear factor in the activation of viral transcription through the major immediate early promoter [89].

**Fig. 3:**
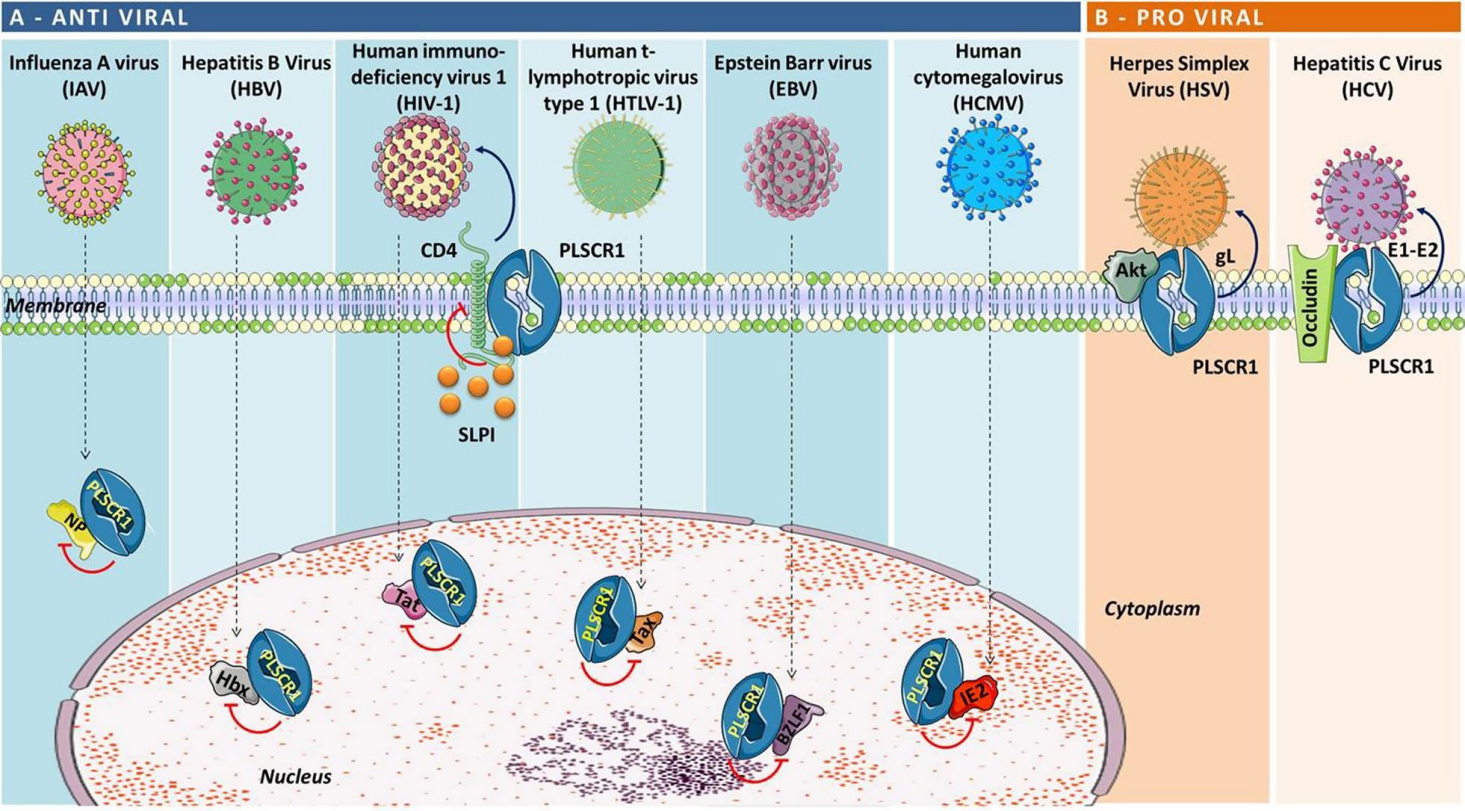
PLSCR1/viral protein interactions. Schematic representation of **A** antiviral activity of PLSCR1 through the interaction with different types of virus and consequent inhibition of viral replication; **B** pro-viral activity of PLSCR1 that, thanks to its localization in the plasma membrane, favours virus entry in the host cell

In summary, these studies documented the ability of PLSCR1 to act as a molecular sponge for a number of viral proteins required at different key points of the viral life cycle, thus sequestering them from their cellular interactors (Fig. 3A).

### Pro-viral activity of PLSCR1

Although the antiviral activity of PLSCR1 is well documented, there is some evidence that PLSCR1 might favor virus propagation as well (Fig. 3B). For example, Cheshenko et al*.* found that PLSCR1 can be activated following the binding of glycoprotein L of herpes simplex virus (HSV) 1 and 2 to the external layer of the cell membrane and the engagement of nectin-1 [90]. Following HSV binding, the release of a small amount of Ca^2+^ near the plasma membrane appeared to be sufficient to activate PLSCR1. PLSCR1 activation could in turn promote PS and Akt flip-flop to the outer leaflet of the membrane where Akt could interact with the viral glycoprotein B, thus favoring HSV entry into the target cell. Indirect or direct PLSCR1 targeting through the addition of a cell-permeable Ca^2+^ chelator or a pharmacological PLSCR1 antagonist, or upon transfection with a PLSCR1 small interfering RNA, impaired Akt externalization and specifically blocked HSV entry [90].

It can be inferred then, that PLSCR1 may exert anti- or pro-viral effects depending on the viral species. There are instances, though, where PLSCR1 has been reported to exert contrasting effects on the same species. For example, while PLSCR1 can contribute to IFN-mediated antiviral activity during HCV infection [30], Gong et al*.* showed that cell surface-localized PLSCR1 may bind to the HCV envelope proteins E1 and E2, thus promoting the initial attachment of HCV onto hepatocytes, possibly by affecting clathrin-mediated endocytosis. As a result, downregulation of PLSCR1 expression inhibited HCV entry and infection [26].

Taken together, these observations strongly suggest that PLSCR1 expression may affect susceptibility to infection through several mechanisms depending on the virus, the host cell type, and/or the interacting protein.

## Conclusions

Extensive research over the past years has unraveled a central role for PLSCR1 in the membrane externalization of PS. However, accumulating evidence indicates that PLSCR1 exerts a complex array of functions, which makes it a challenging protein to study.

We have herein provided an overview of the current knowledge about PLSCR1 cellular functions, with a particular focus on its ability to interact with endogenous and exogenous proteins. The studies discussed above have been performed by using widely disparate experimental models, most of which in vitro, such that it is hard to reconcile PLSCR1 multiple functions, often times controversial, and fully delineate its overall impact on cell physiology and disease.

In addition, although it is well known that PLSCR1 can be found in different cellular compartments, how its distribution may influence its activity is not yet completely understood. It is now apparent that PLSCR1 is involved in multiple cellular processes besides PS externalization and apoptosis, depending on the functions of its interactors and on whether these interactions lead to their inhibition or activation. Given the extreme diversity of PLSCR1 multiple functions, more comprehensive studies, and appropriate pre-clinical or clinical models, are required to thoroughly understand the fundamental biology of PLSCR1.

Based on current evidence, it is difficult to predict when PLSCR1 overexpression or inhibition would be beneficial in human disease since PLSCR1 function may vary according to cell types and disease processes. Nevertheless, a number of reports have highlighted the possible beneficial effects of PLSCR1 targeting in colorectal cancers, hepatic cancers, and lung adenocarcinoma, where blockade or silencing of this protein resulted in the inhibition of tumor growth and metastasis [19, 79, 80, 91].

As discussed above, the interactions of PLSCR1 with a host of viral proteins may either promote or antagonize viral functions depending on the viral species, the cell type and the specific interactor. As PLSCR1 is promptly induced following type I IFN signaling, and in turn contributes to maintaining this pathway active, it would be quite informative to monitor PLSCR1 activity throughout different phases of a viral infection. However, evidence to date is sufficient to at least speculate that PLSCR1 association with viral proteins within the nucleus may exert antiviral effects by affecting viral replication, whereas its interaction with viral proteins at the plasma membrane may rather boost viral infection by facilitating virus entry. More studies are warranted to provide conclusive evidence for these associations and understand their significance. The elucidation of the mechanisms that regulate PLSCR1 expression, along with the identification of the signaling pathways in which PLSCR1 is involved, may lead to the development of future pharmacological strategies aimed at targeting this factor in cancer, viral infections, allergy and autoimmunity, and possibly other conditions.

## Data Availability

Not applicable.

## Abbreviations

AD: Alzheimer’s disease
AIF: Apoptosis-inducing factor
ANCA: Anti-neutrophil cytoplasm autoantibodies
ANG: Angiogenin
APP: Amyloid precursor protein
ATG: Autophagy-related protein
ΒACE: β-Secretase β-site amyloid precursor protein (APP)-cleaving enzyme
BLBC: Basal-like breast cancer
CBP: CREB-binding protein
CpG ODN: CpG motif-containing oligodeoxynucleotides
CREB: cAMP-responsive element-binding protein
EBV: Epstein-Barr virus
EGFR: Epidermal growth factor receptor
FcεRI: High-affinity receptor for immunoglobulin E
GPA: Granulomatosis with polyangiitis
HBV: Hepatitis B virus
HCMV: Human cytomegalovirus
HCV: Hepatitis C virus
HIV-1: Human immunodeficiency virus 1
HSV: Herpes simplex virus
HTLV-1: Human t-lymphotropic virus type 1
IAV: Influenza A virus
IE2: HCMV immediate early protein 2
IFN: Interferon
IP3R1: Inositol 1,4,5-triphosphate receptor type 1
IRF: Interferon regulatory factor
JNK: C-Jun N-terminus kinase
LAT: Linker for activation of T-cells
MK: Midkine
MV: Microvesicle
NLS: Nuclear localization signal
NP: Nucleoprotein
NPC: Nasopharyngeal carcinoma
OSR1: Oxidative-stress responsive 1
pDC: Plasmocytoid dendritic cell
PE: Phosphatidylethanolamine
PKCδ: Protein kinase Cδ
PLSCR1: Phospholipid scramblase 1
PR3: Proteinase 3
PS: Phosphatidylserine
RELT: Receptor expressed in lymphoid tissues
SLE: Systemic lupus erythematosus
SLPI: Secretory leucocyte protease inhibitor
SPAK: SPS1-related proline/alanine-rich kinase
STAT: Signal transducer and activator of transcription
TAX: HTLV-1 transactivator protein
TLR9: Toll like receptor 9
TOPO: Topoisomerase
TRPC5: Transient receptor potential canonical 5
